# Organ‐Specific Shifts in Aerobic and Anaerobic Metabolism Throughout Metamorphosis Into Adulthood in a Fully Aquatic Amphibian

**DOI:** 10.1096/fj.202502054R

**Published:** 2025-09-10

**Authors:** Neal J. Dawson, Miguel Hernandez‐Gonzalez, Pat Monaghan, Neil B. Metcalfe, Pablo Burraco

**Affiliations:** ^1^ School of Biodiversity, One Health and Veterinary Medicine, Graham Kerr Building, College of Medical, Veterinary and Life Sciences University of Glasgow Glasgow UK; ^2^ Behavioural Ecology and Ecophysiology Group, Department of Biology University of Antwerp Wilrijk Belgium; ^3^ Doñana Biological Station (CSIC) Seville Spain

**Keywords:** citrate synthase, cytochrome c oxidase, developmental physiology, energetic remodeling, lactate dehydrogenase, life transitions, metabolic capacity, succinate dehydrogenase

## Abstract

Most animals experience abrupt developmental transitions involving major tissue remodeling, but the links with metabolic changes remain poorly understood. We examined ontogenetic changes in mitochondrial volume, oxidative capacity, oxygen consumption capacity, and anaerobic capacity across four organs (gut, liver, heart, and hindlimb muscle) in *Xenopus laevis* from metamorphosis to adulthood. These organs differ in the extent of developmental transformation. Mitochondrial volume increased notably in the metamorphosing gut and remained stable in the heart, decreased in the liver, and increased in the hindlimb muscle post‐metamorphosis. Oxidative capacity was lower at metamorphosis than in later stages in the gut, heart, and hindlimb and showed the opposite pattern in the liver. Oxygen consumption capacity remained stable in the gut and liver but increased in the post‐metamorphic heart and hindlimb. Anaerobic capacity increased with age across all organs. These findings reveal organ‐specific patterns in metabolic capacity during development, reflecting varying energetic demands such as tissue remodeling during metamorphosis (e.g., in the gut) or increased locomotion post‐metamorphosis (e.g., the heart and hindlimb muscle). Higher anaerobic capacity suggests an alternative way to cope with low oxygen during intense activity post‐metamorphosis. This work provides a foundation for understanding how metabolic dynamics shape developmental transitions and their eco‐evolutionary implications.

## Graphical abstract

We examined organ‐specific metabolic changes during development in the frog *Xenopus laevis* . Mitochondrial volume, oxidative capacity, and oxygen consumption capacity increased with age in the gut, heart, and hindlimb muscle, but these processes varied across organs and were shaped by the metamorphic process (e.g., in the gut and the hindlimb muscle). In the liver, aerobic metabolism decreased across development. Anaerobic capacity increased consistently across organs with age. These findings highlight the organ‐specific nature of energy metabolism during developmental and life‐history transitions.

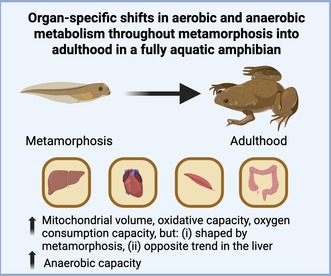

## 1 Introduction

The study of how the mode and efficiency of energy production changes during early life is of central interest to understand the metabolic machinery governing developmental processes in animals [1, 2]. So far, the bulk of the available information on contributions of aerobic and anaerobic metabolism to animal development comes from species in which body structures mostly develop early in life [1, 3, 4, 5, 6]. However, the life cycle of more than three quarters of known animal species includes an abrupt developmental transition, termed metamorphosis [7]. Metamorphosis is an energy‐expensive process that mediates the transition between two or more life stages where organisms undergo substantial transformations in their morphology, behavior, and physiology, often accompanied by a change in habitat [8]. Investigating key energy producing pathways from early life through adult stages in metamorphosing species can therefore provide important insights into the mechanisms mediating major life stage transitions, particularly under the current global change scenario [7, 9, 10, 11].

Mitochondrial respiration consists of the oxidation of nutrients such as glucose or fatty acids, to produce ATP which is used as energy to drive most cellular processes [1]. The efficiency and capacity of mitochondrial respiration will ultimately dictate the contribution of aerobic metabolism to the development of different body structures. The abundance of mitochondria is often positively correlated with energy production and performance [12] and, therefore, a higher volume of mitochondria is expected to be found in tissues such as the skeletal muscle or liver that have a sustained requirement for greater amounts of energy (e.g., [13]). However, ATP can also be generated without oxygen via anaerobic metabolism. This is particularly important in tissues with short‐term energy demands that outstrip the supply of oxygen for oxidative phosphorylation, such as the skeletal muscle during short‐duration, high‐intensity exercise [2]. Hence, it is expected that the aerobic and anaerobic capacity of cells, tissues, and organs will depend on the nature of their energetic requirements, and this balance is likely to change over ontogeny. However, this has been little studied, particularly in species undergoing metamorphosis, where tissues are expected to experience major shifts in energy demands [14, 15, 16, 17].

Among vertebrates, amphibians undergo the most drastic developmental changes. The life cycle of many amphibians involves metamorphosis from a herbivorous tadpole to a carnivorous frog [18]. Within a few days, several body structures experience partial or complete remodeling, whereas others are newly formed or resorbed. These processes involve extensive cell growth, de novo formation of stem cells, and apoptosis [18]. Amphibian metamorphosis is thought to be metabolically costly [7] as it involves the induction of cellular oxidative stress or substantial reductions in fat reserves [19, 20]. Changes in the amount of aerobic and anaerobic metabolism are therefore expected to vary throughout metamorphosis in response to the changing energy requirements needed to support the developmental processes, and these changes will differ among tissues. Also, energy demands may differ across organs during post‐metamorphic stages, with emphasis on species with indeterminate growth in which organ function and size continue to change through adulthood.

Here, we investigate the activity of key metabolic enzymes, acting as biomarkers for aerobic and anaerobic metabolic capacity in four organs across different life stages in a fully aquatic amphibian ( *Xenopus laevis* ). We collected samples at five stages of development from the larva through to the sexually mature adult. At each sampling point, we sampled four different organs: the gut, liver, heart, and hindlimb muscle. These organs differ in their degree of remodeling across development. As in other anurans, the gut of *Xenopus laevis* is extensively remodeled during metamorphosis, including shortening by ca. 70%, apoptosis of epithelial cells, and the development of the adult (carnivorous) intestine from stem cells [21, 22]. The development of hindlimbs takes place during the larval period and, from metamorphosis onwards, there is a major development of new tissue as larval primary muscle myofibers are progressively replaced by secondary adult multinucleated myofibers [23]. Liver and heart experience a much less dramatic transformation but greatly increase in size from larval to the adult stage; they are expected to have major metabolic demands due to their roles in functions such as detoxification and regulation of metabolic processes in the case of the liver, and oxygen transport essential for growth and behavioral activities in the case of the heart [24, 25].

The aerobic and anaerobic capacity was assessed in each organ by measurements of key enzyme activities from each of these energy producing processes. In this study, we refer to “capacity” (i.e., oxidative capacity, oxygen consumption capacity, or anaerobic capacity) because we estimated the maximum amount of product that can be produced by the different enzymes measured in this study. First, we measured the activity of citrate synthase (CS), an enzyme responsible for the first reaction of the Krebs cycle in the mitochondrial matrix [26]; CS activity is considered a good proxy for mitochondrial volume [12, 27]. The activity of two enzymes, succinate dehydrogenase (SDH, complex II) and cytochrome c oxidase (COX, complex IV), is used as indicators of oxidative capacity and of oxygen consumption capacity of the mitochondria, respectively [28, 29]. SDH is referred to as a marker of oxidative capacity because it plays a major role in both the Krebs cycle and the electron transport chain (ETC). It feeds electrons into the ETC via oxidation of flavin adenine dinucleotide (FADH_2_) which is generated by the oxidation of succinate. COX is referred to as oxygen consumption capacity because it catalyzes the last step of the ETC, the reduction of oxygen to generate water. Therefore, COX activity may provide insights into the ability of mitochondria to keep consuming oxygen and sustain oxidative phosphorylation [12]. Finally, we estimated anaerobic capacity through the measurement of lactate dehydrogenase activity (LDH) because this enzyme plays a key role in the conversion of lactate to pyruvate, a process that is highly active during anaerobic metabolism [30]. Our aim in this study is to examine organ‐specific changes in aerobic and anaerobic capacity across life stages. We hypothesized that organs undergoing extensive transformation during metamorphosis (i.e., the gut and the hindlimb muscle) would show an enhancement of both aerobic and anaerobic metabolism throughout the metamorphic transition. We also predicted that organs such as the heart and the hindlimb muscle, that have sustained energetic demands post‐metamorphosis, would develop higher levels of aerobic and anaerobic capacity to meet the complex energy demands linked to locomotion. Finally, organs experiencing large increases in size but no major developmental changes nor more intense functional demands, i.e., the liver would not experience much variation in their aerobic and anaerobic capacity over ontogenetic transitions.

## 2 Material and Methods

We obtained five wild‐type clutches from the European *Xenopus* Resource Centre (University of Portsmouth, United Kingdom). All embryos were maintained at 19°C (which is also the temperature used at the provider's facilities) with a light: dark cycle of 12:12 h and a water circulation system to provide filtration. Once hatched, groups of 18 randomly selected larvae per clutch were placed into three separate tanks (initial density of 18 larvae/tank, 5 clutches, 15 tanks in total). We collected samples at five developmental stages. Two sampling points covered metamorphosis: at the onset (i.e., when forelimbs are already visible and tail resorption starts), and at the end of metamorphosis (i.e., when forelimbs appear and tail is fully resorbed, respectively), described by Nieuwkoop and Faber (NF) as NF60 and NF66 stages [31]. The other three sampling points were chosen to be representative of three post‐metamorphosis stages: 70‐day‐old froglets (~13 days after completion of metamorphosis in our experiment, when metamorphic transformations are thought to be completed), 7‐month‐old frogs (i.e., juveniles during a phase of extensive growth and approaching sexual maturity), and 2‐year‐old frogs (i.e., sexually mature adults that had reached average adult body size for laboratory specimens in this species) [32, 33]. Frog density was reduced to three individuals/tank after the 70‐day sampling point, and this density was maintained until frogs were 7 months old. At this point, we randomly selected one individual per tank to be retained and housed these selected frogs in a single 100‐L tank until the end of the study (i.e., *N* = 15 frogs in this tank) (for further details, see [34, 35]). We fed larvae ad libitum with a 1:1 mix of Spirulina and Sera Micro powders diluted in water, and frogs ad libitum with sinking trout pellets. At the time of sampling, individuals were euthanized by immersion in a 2 g/L of tricaine methanesulfonate (Sigma‐Aldrich) buffered to pH 7.0 with sodium bicarbonate, following the humane methods approved by the UK Home Office. Their body mass was then recorded (to 0.001 g) and samples were collected of heart, liver, gut, and hindlimb muscle. All tissues were stored at −80°C until assayed.

### 2.1 Enzymatic Assays

Enzymatic activities were quantified following the protocols developed by [36] and optimized for *Xenopus* organs. Collected organs were held on ice and mechanically homogenized in homogenization buffer [100 mM KH_2_PO_4_ buffer at pH 7.2, containing 1 mM EGTA, 1 mM EDTA, and 1 mM phenylmethylsulfonylfluoride (PMSF)]. The resulting homogenates were centrifuged at 1000 g for 3 min, and then the supernatant was collected. The activity of each enzyme was assayed independently by adding 10 μL of homogenate into a reaction well with the specific reagents and monitoring the rate of disappearance of an optically active compound using spectrophotometry (details are indicated below). All samples were randomized (for both developmental stage and organ) across the assay plates and run for 30 min in triplicates with their respective blanks using a SpectraMaxPlus 384 spectrophotometer (Molecular Devices, San Jose, California, USA) at 21°C (controlled room temperature of the laboratory). Data were recorded and analyzed using the coupled SoftMax Pro 6.3 software. Activity graphs were individually and manually processed to record the slope of the reaction over the linear range (i.e., change in optical density, Δ O.D.). Δ O.D. values were converted into enzyme activity following [36], to account for the extinction coefficient of each enzyme (*ε*), the path length of the reaction well, and the dilution factor of the homogenate in the case of lactate dehydrogenase (1:10). Sample sizes for the different organs and developmental stages are shown in Table S1–S6.

CS assays [*ε* = 14.15 mM^−1^ cm^−1^] were run in 100 mM KH_2_PO_4_ buffer (pH 8.0) by addition of 0.15 mM acetyl‐CoA, 0.15 mM 5,5′‐dithiobis‐2‐nitrobenzoic acid, and 0.5 mM oxaloacetate (omitted in blank). The reaction was monitored at a wavelength of 412 nm.

SDH assays [*ε* = 21.9 mM^−1^ cm^−1^] were run in 100 mM KH_2_PO_4_ buffer (pH 8.0) by addition of 0.3 mM KCN, 0.05 mM decylubiquinone, 0.05 mM 2,6‐dichlorophenolindophenol, and 20 mM succinate (omitted in control). The reaction was monitored at 600 nm.

COX assays [*ε* = 28.5 mM^−1^ cm^−1^] were run in 100 mM KH_2_PO_4_ buffer (pH 8.0) by the addition of 0.2 mM reduced cytochrome c, and the omission of homogenates in the blanks. The reaction was monitored at 550 nm.

LDH assays [*ε* = 6.22 mM^−1^ cm^−1^] were run in 100 mM KH_2_PO_4_ buffer (pH 7.2) by addition of 0.15 mM NADH and 2.5 mM pyruvate (omitted in control). The reaction was monitored at a wavelength of 340 nm.

### 2.2 Statistical Analyses

Analyses were conducted in R software (version 4.2.3) [37]. First, we checked for overall differences in CS activity across organs and developmental stages by running a linear mixed model that included CS activity as the dependent variable, developmental stage and organ and their interaction as independent variables, body mass as a covariate, and individual and CS plate as the random factors. In that model, the interaction between developmental stage and organ was significant (Table S2) whereas the effect of body mass was nonsignificant (Table S2). Based on that first exploratory model, we conducted reduced linear models for each organ, using CS activity as the dependent variable, developmental stage as the independent variable, and CS plate as the random factor.

We then investigated whether the activity of two enzymes used as markers of oxidative capacity (SDH activity) and mitochondrial oxygen consumption capacity (COX activity) varied across organs and developmental stages. Since both are mitochondrial enzymes, SDH and COX activities were corrected for mitochondrial volume by including CS activity as a covariate in the models (*R* ^2^ for the correlations between SDH and CS, and COX and CS were 0.27 and 0.28, respectively). We first conducted a linear mixed model including either SDH or COX activity as the dependent variable, developmental stage, organ, and their interaction as independent variables, and body mass and CS activity as covariates; and individual ID, and SDH or COX plate, and CS plate, as random factors. Since the interaction between organ and developmental stage was significant in both models (Table S2), and the effect of body mass was not significant (Table S2), we conducted reduced models for each enzyme and organ including either SDH or COX activity as the dependent variable, developmental stage as the independent variable, CS activity as the covariate, and CS plate and either SDH or COX plate as the random factors.

Finally, we investigated whether the activity of LDH, a marker of anaerobic capacity, varies across organs and developmental stages. Anaerobic respiration occurs in the cell cytoplasm, which explains why the relationship between LDH and CS activity was negligible (*R* ^2^ = 0.022). We therefore did not include CS activity in LDH activity models. We first conducted a full model including LDH activity as the dependent variable, developmental stage and organ and their interaction as independent variables, body mass as the covariate, and individual ID and LDH plate as the random factors. Since in that model the interaction between organ and developmental stage was significant whereas the effect of body mass was not (Table S2), we conducted reduced models for each organ including LDH activity as the dependent variable, developmental stage as the independent variable, and LDH plate as the random factor.

When the effect of developmental stage was significant, we conducted post hoc tests using false discovery rate adjustments. Enzyme activities were log‐transformed to meet parametric assumptions.

## 3 Results

**FIGURE 1:**
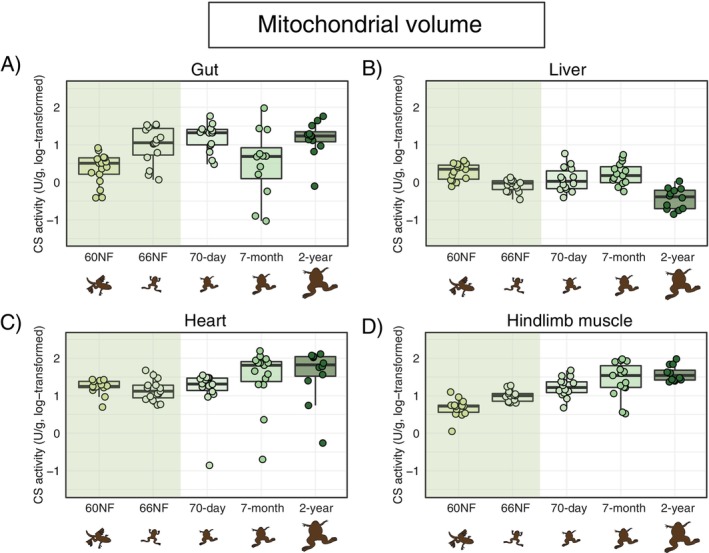
Mitochondrial volume (estimated through citrate synthase—CS—activity, in U/g tissue wet weight) across organs and developmental stages of *Xenopus laevis* . Developmental stages are: NF60 (start of metamorphosis), NF66 (end of metamorphosis), 70 days old (~13 days after metamorphosis), 7 months old, and 2 years old. Boxplots indicate the interval between first and the third quartiles, black lines show the median value, bars depict minimum and maximum values within 1.5 interquartile range. The green band highlights the period of metamorphosis. The *x*‐axis and drawings are non‐scale representations of time and appearance of individuals, respectively. Pairwise post hoc comparisons among developmental stages are provided in Table S3.

Mitochondrial volume (estimated via CS activity) varied across organs and developmental stages (Figure 1A; Table 1; Table S3). In the gut, mitochondrial volume increased throughout metamorphosis (i.e., between NF60 and NF66), remained relatively stable until day 70, then decreased in 7‐month‐old frogs before increasing again in 2‐year‐old adults (Figure 1A; Table 1; Table S3). In the liver, mitochondrial volume decreased throughout metamorphosis, remained unchanged until frogs were 7 months old, and then decreased further in 2‐year‐old frogs (Figure 1B; Table 1; Table S3). In the heart, mitochondrial volume did not change across developmental stages (Figure 1C; Table 1). Finally, in the hindlimb muscle, mitochondrial volume gradually increased from metamorphosis to 7 months and then remained stable (Figure 1D; Table 1; Table S3).

**TABLE 1:** Linear mixed models testing for the effect of developmental stage on mitochondrial volume (through citrate synthase—CS—activity), oxidative capacity (through succinate dehydrogenase—SDH—activity), oxygen consumption capacity (through cytochrome oxidase c—COX—activity), and anaerobic capacity through (lactate dehydrogenase—LDH—activity) in the gut, liver, heart, and hindlimb of *Xenopus laevis* .

		Gut			Liver			Heart			Hindlimb muscle		
		Chi‐sq	df	*p*	Chi‐sq	df	*p*	Chi‐sq	df	*p*	Chi‐sq	df	*p*
Mitochondrial volume	Developmental stage	18.46	4	0.001	42.72	4	< 0.001	6.67	4	0.145	55.22	4	< 0.001
Oxidative capacity	Developmental stage	26.88	4	< 0.001	11.01	4	0.026	158.33	4	< 0.001	20.57	4	< 0.001
	Citrate synthase activity	17.28	1	< 0.001	0.88	1	0.349	41.36	1	< 0.001	12.38	1	< 0.001
Oxygen consumption capacity	Developmental stage	2.53	4	0.639	14.17	4	0.007	55.12	4	< 0.001	3.93	4	0.416
	Citrate synthase activity	17.29	1	< 0.001	2.05	1	0.153	8.65	1	0.003	16.00	1	< 0.001
Anaerobic capacity	Developmental stage	18.46	4	0.001	42.72	4	< 0.001	6.67	4	0.145	55.22	4	< 0.001

*Note:* Values of oxidative capacity (succinate dehydrogenase activity) and oxygen consumption capacity (cytochrome oxidase c) were corrected for mitochondrial volume (citrate synthase activity). In all models assay plate number of the enzyme was included as a random factor.

**FIGURE 2:**
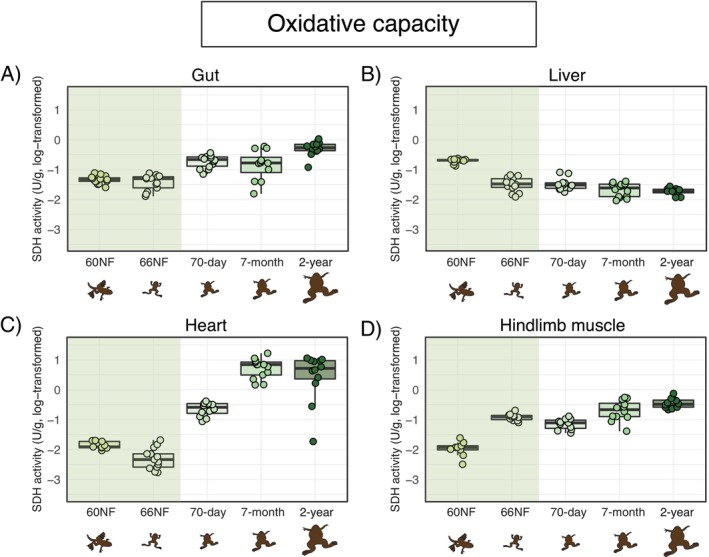
Oxidative capacity (estimated through succinate dehydrogenase—SDH—activity, in U/g tissue wet weight) across organs and developmental stages of *Xenopus laevis* . Developmental stages as in Figure 1. Boxplots indicate the interval between first and the third quartiles, black lines show the median value, bars depict minimum and maximum values within 1.5 interquartile range. The green band highlights metamorphosis. The *x*‐axis and drawings are non‐scale representations of time and appearance of individuals, respectively. Pairwise post hoc comparisons among developmental stages are provided in Table S4.

Oxidative capacity (estimated via SDH activity and normalized to CS activity to account for changes in mitochondrial volume) varied across organs and developmental stages (Figure 2; Table 1; Table S4). In the gut, oxidative capacity was lower at the onset and the end of metamorphosis than later in life (Figure 2A; Table S4). In contrast, in the liver, oxidative capacity the liver was highest at the onset of metamorphosis (NF60) than later in life (Figure 2B; Table S4). In the heart, oxidative capacity was remarkably lower at metamorphic than at post‐metamorphic stages, increased in 70‐day‐old juveniles, and then it remained unchanged in 7‐month and 2‐year frogs (Figure 2C; Table S4). Finally, in the hindlimb muscle, oxidative capacity increased from the onset to the end of metamorphosis, then remained at the higher level into adulthood (Figure 2D; Table S4).

**FIGURE 3:**
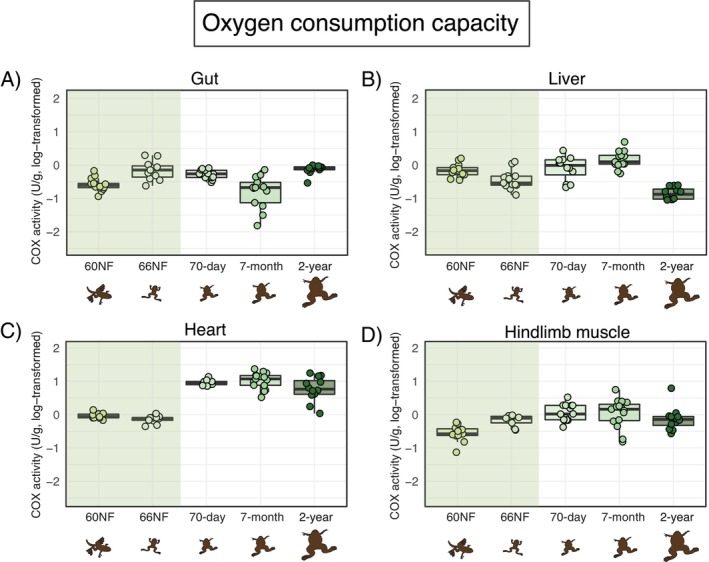
Oxygen consumption capacity (estimated through cytochrome c oxidase—COX–activity, in U/g tissue wet weight) across organs and developmental stages of *Xenopus laevis* . Developmental stages as in Figure 1. Boxplots indicate the interval between first and the third quartiles, black lines show the median value, bars depict minimum and maximum values within 1.5 interquartile range. The green band highlights metamorphosis. The *x*‐axis and drawings are non‐scale representations of time and appearance of individuals, respectively. Pairwise post hoc comparisons among developmental stages are provided in Table S5.

Oxygen consumption capacity (estimated via COX activity, once corrected for mitochondrial volume) was again dependent on the organ and developmental stage (Figure 3; Table 1; Table S5). In the gut, oxygen consumption capacity did not vary across developmental stages (Figure 3A; Table 1). In contrast, in the liver, oxygen consumption capacity remained stable throughout metamorphosis, increased in 70‐day‐old and 7‐month‐old frogs, and experienced a marked decrease in 2‐year‐old frogs (Figure 3B; Table S5). In the heart, oxygen consumption capacity was stable throughout metamorphosis and then increased, remaining stable again across the three post‐metamorphic stages (i.e., 70‐day, 7‐month, and 2‐year frogs; Figure 3C; Table S5). Finally, in the hindlimb muscle, oxygen consumption capacity did not vary across ontogeny (Figure 3D; Table 1).

**FIGURE 4:**
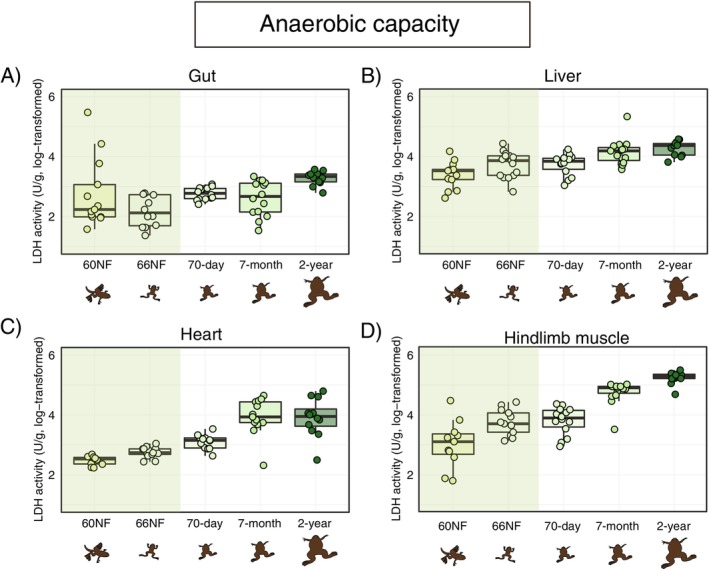
Anaerobic capacity (estimated through lactate dehydrogenase—LDH—activity, in U/g tissue wet weight) across organs and developmental stages of *Xenopus laevis* . Developmental stages as in Figure 1. Boxplots indicate the interval between first and the third quartiles, black lines show the median value, bars depict minimum and maximum values within 1.5 interquartile range. The green band highlights metamorphosis. The *x*‐axis and drawings are non‐scale representations of time and appearance of individuals, respectively. Pairwise post hoc comparisons among developmental stages are provided in Table S6.

Anaerobic capacity (estimated via LDH activity) showed significant variation across organ and developmental stages, the temporal pattern being broadly similar, with a gradual and very consistent increase in activity from metamorphic to adult stages and only minor differences in this trend between organs (Figure 4; Table 1; Table S6).

## 4 Discussion

We demonstrate here that the capacity to produce energy via aerobic and anaerobic metabolism varies considerably across life transitions and between organs of an amphibian undergoing metamorphosis. Increases in aerobic metabolism throughout metamorphosis could be explained by increases in the energy demands of processes like tissue remodeling and transformation, such as in the gut and hindlimb. After metamorphosis, the need for high energy supply for functions such as locomotion, as in the heart and hindlimb muscles, could be behind enhanced aerobic metabolism in these organs. This contrasts with the decrease in aerobic metabolism across life stages in the liver, likely driven by the scant remodeling during metamorphosis and less energy‐demanding processes in post‐metamorphic stages. Likewise, anaerobic capacity increases with age across all organs, with the most pronounced occurrence of this phenomenon in the heart and hindlimb muscle, possibly linked to explosive exercise needs for feeding and antipredator responses. Overall, our study suggests adaptive organ‐specific responses to changing metabolic demands as the organism develops and matures.

The dramatic cellular and tissue remodeling and transformation that take place during metamorphosis are thought to be energetically demanding in themselves [7, 38, 39]. Our study supports and extends this idea, as the extent of remodeling processes seems to be linked to changes in both aerobic and anaerobic metabolism between organs. In the gut, extensive cell apoptosis and stem cell dedifferentiation drive the formation of intestinal folds during metamorphosis [21, 22]. We found that gut mitochondrial volume and oxygen consumption capacity increased from the onset to the end of metamorphosis, most probably to provide the energy required for remodeling. Intriguingly, anaerobic capacity decreased in the gut during metamorphosis—contrasting with the changes observed in the other organs—potentially due to shifts in the composition of gut microbiota [40]. Particularly, this may be related to changes in gut microbial composition across development, which can alter local oxygen levels and influence LDH expression and activity [40]. In the hindlimb muscle, we observed increases in aerobic metabolism (i.e., higher mitochondrial volume and oxidative capacity) during metamorphosis. At this life transition, multinucleated muscle fibers are formed from amitotic division of primary nuclei [23], a process that appears to require the remodeling of their aerobic demands. In contrast, in the metamorphosing liver and heart, we observed either decreases or no changes in aerobic metabolism. These two organs do not undergo dramatic remodeling or transformation during metamorphosis, while behavioral activity declines and feeding ceases [18], which may contribute to a reduced metabolic workload and lower energy demands. Finally, in the hindlimb muscle, liver, and heart, anaerobic capacity increased throughout metamorphosis, likely acting as an alternative pathway to aerobic production of energy during the metamorphic transition and as the organism matures.

From metamorphosis onwards, some amphibian species, such as *Xenopus laevis* , become carnivorous and experience considerable increases in growth until adulthood (~40 fold increase in body mass between 70‐day‐old vs 2‐year‐old individuals in our study). Overall, adult frogs (7‐month‐old and 2‐year‐old individuals) had a higher aerobic capacity than metamorphosing larvae, which is particularly notable in the heart and hindlimb muscle, two organs that experience contrasting developmental processes in *Xenopus laevis* . Heart development is completed during the larval phase [41], and thus our findings may suggest a remodeling of the post‐metamorphic heart mitochondria phenotype. These changes are in accordance with the observed maturation of mitochondrial processes in the heart of different vertebrate species (e.g., pigs, rats, and humans [42]). In the hindlimb muscle, variation in cell population (i.e., proportion of myofiber types across development) may be behind changes in its post‐metamorphic aerobic metabolism. Also, both in the heart and hindlimb muscle, anaerobic capacity was much higher at adulthood (i.e., 7‐month and 2‐year old frogs) than earlier in life (i.e., at and immediately after metamorphosis). In anaerobic metabolism, glycogen or glucose metabolism still produces pyruvate that can undergo either oxidative phosphorylation at the mitochondria or can be reversibly converted to lactate within the cell cytoplasm when oxygen levels are not able to sustain oxidative phosphorylation [43]. High anaerobic capacity in the heart and the hindlimb muscle could, therefore, be changing in accordance with high lactate production (an energy‐rich postexercise molecule) in the hindlimb muscle through glycolysis at adulthood, when high energy supply is needed to support activities such as swimming, foraging, or antipredatory behaviors [44, 45, 46, 47]. However, the higher activity of LDH in the heart will likely contribute to greater lactate consumption, as exogenous lactate is preferentially oxidized in the heart [48]. In the liver, aerobic capacity decreased with age, while anaerobic metabolism gradually increased from metamorphosis until adulthood. Large amounts of lactate can be transported by the blood to the liver where it is converted into glucose via gluconeogenesis, which can then be recirculated to the muscles for use [49]. Finally, the gut of post‐metamorphic frogs experienced increases in both oxidative capacity and anaerobic capacity. It is important to highlight here that we estimated oxidative capacity through the activity of the enzyme succinate dehydrogenase, a key enzyme involved in succinate metabolism, which is implicated in the regulation of host–microbiome interactions [50] and promotion of the colonization of strict anaerobes, which represent 99% of the gut microbiota [51]. Since succinate also plays a vital role in regulating other pathways in the gut, such as the function of the mucosal immune cells [50], this might explain the observed variation in the activity of this enzyme across the development of the gut as the immune system and microbiome are changing [52].

These organ‐specific shifts in energy metabolism across developmental stages highlight the importance of metabolic reprogramming as a key component of life‐history transitions. The metamorphic process in amphibians involves not only morphological and functional transformations but also a profound reorganization of energy production strategies tailored to the demands of each life stage. While our findings may reflect the aquatic lifestyle of *Xenopus laevis* , similar metabolic reprogramming during metamorphosis is likely to occur in anurans with terrestrial adult phases and potentially could be extended to other taxa undergoing major developmental transitions, such as fish or insects. Yet, terrestrial organisms may experience additional selective pressures such as dehydration stress or gravity effects on locomotion, which may further shape post‐metamorphic metabolism. Aerobic and anaerobic pathways are differentially modulated depending on the degree of organ remodeling, developmental timing, and expected post‐metamorphic function. Such plasticity in metabolic traits likely reflects adaptive responses that optimize performance in changing ecological contexts, like the shift from a relatively sedentary, herbivorous aquatic environment to a more mobile, carnivorous adult. This supports the view that developmental transitions are windows of heightened physiological change where metabolic flexibility enables organisms to realign organ functions with new behavioral and ecological demands. However, we acknowledge that the lack of direct functional measures limits interpretation and encourage future studies to link enzyme activity with organismal performance.

## Author Contributions

Neal J. Dawson, Miguel Hernandez‐Gonzalez, Pat Monaghan, Neil B. Metcalfe, and Pablo Burraco designed research; Neal J. Dawson, Miguel Hernandez‐Gonzalez, and Pablo Burraco performed the experiment and laboratory assays; Pablo Burraco analyzed the data; Neal J. Dawson, Miguel Hernandez‐Gonzalez, Pat Monaghan, Neil B. Metcalfe, and Pablo Burraco wrote the paper.

## Ethics Statement

Since this experiment did not cause stress, pain, or lasting harm to the animals, the University's Named Veterinary Surgeon informed us in writing that it did not require a Home Office license for animal experimentation.

## Conflicts of Interest

The authors declare no conflicts of interest.

## Supporting information


**Tables S1–S6:** fsb271035‐sup‐0001‐TableS1‐S6.docx.

## Data Availability

Data supporting this article are available at https://figshare.com/s/78d1df4a17e32bcf0683?file=55085618.
