# Supplementary material for: Organ‐Specific Shifts in Aerobic and Anaerobic Metabolism Throughout Metamorphosis Into Adulthood in a Fully Aquatic Amphibian
Source: FASEB J. 2025 Sep 10;39(18):e71035. doi: 10.1096/fj.202502054R (PMC12423575; doi:10.1096/fj.202502054R)
Supplement: Supplementary file 1 — Tables S1–S6: fsb271035‐sup‐0001‐TableS1‐S6.docx. [file FSB2-39-e71035-s001.docx]

**SUPPLEMENTARY MATERIAL OF**

**Organ-specific shifts in aerobic and anaerobic metabolism throughout metamorphosis into adulthood in a fully aquatic amphibian**

**Table S1.** Sample size across developmental stages, tissues and enzymes measured in *Xenopus laevis*.

|  | **Mitochondrial volume**  **(citrate synthase)** | | | | |
| --- | --- | --- | --- | --- | --- |
|  | **60NF** | **66NF** | **70-day** | **7-month** | **2-year** |
| **Gut** | 15 | 15 | 14 | 15 | 12 |
| **Liver** | 15 | 15 | 14 | 15 | 12 |
| **Heart** | 13 | 15 | 14 | 15 | 12 |
| **Hinblimb muscle** | 14 | 14 | 14 | 15 | 12 |
|  | **Oxidative capacity**  **(succinate dehydrogenase)** | | | | |
|  | **60NF** | **66NF** | **70-day** | **7-month** | **2-year** |
| **Gut** | 15 | 13 | 14 | 12 | 12 |
| **Liver** | 13 | 14 | 12 | 14 | 11 |
| **Heart** | 9 | 13 | 12 | 13 | 12 |
| **Hinblimb muscle** | 9 | 11 | 11 | 12 | 12 |
|  | **Oxygen consumption capacity (citochrome c oxidase)** | | | | |
|  | **60NF** | **66NF** | **70-day** | **7-month** | **2-year** |
| **Gut** | 14 | 14 | 13 | 13 | 12 |
| **Liver** | 13 | 13 | 12 | 13 | 12 |
| **Heart** | 10 | 12 | 9 | 15 | 12 |
| **Hinblimb muscle** | 11 | 9 | 14 | 15 | 12 |
|  | **Anaerobic capacity**  **(lactate dehydrogenase)** | | | | |
|  | **60NF** | **66NF** | **70-day** | **7-month** | **2-year** |
| **Gut** | 13 | 13 | 14 | 13 | 12 |
| **Liver** | 13 | 15 | 14 | 15 | 11 |
| **Heart** | 10 | 13 | 12 | 13 | 12 |
| **Hinblimb muscle** | 11 | 13 | 14 | 15 | 12 |

**Table S2.** Summary of the full linear mixed models testing for the effect of developmental stage, organ type, and their interaction, on mitochondrial volume (citrate synthase activity), oxidative capacity (succinate dehydrogenase activity), oxygen consumption capacity (cytochrome oxidase c), and anaerobic capacity (lactate dehydrogenase) of *Xenopus laevis*. Values of succinate dehydrogenase and cytochrome c oxidase were corrected for citrate synthase activity. In all the models, body mass was included as a covariate and the assay plate of each enzyme was included as a random factor.

|  | **Citrate synthase** | | | **Succinate dehydrogenase** | | | **Cytochrome c oxidase** | | | **Lactate dehydrogenase** | | |
| --- | --- | --- | --- | --- | --- | --- | --- | --- | --- | --- | --- | --- |
|  | **Chi-sq** | **Df** | **P-value** | **Chi-sq** | **Df** | **P-value** | **Chi-sq** | **Df** | **P-value** | **Chi-sq** | **Df** | **P-value** |
| **Developmental stage** | 4.61 | 4 | 0.330 | 17.76 | 4 | 0.001 | 23.58 | 4 | <0.001 | 73.99 | 4 | <0.001 |
| **Organ** | 293.36 | 3 | <0.001 | 30.64 | 3 | <0.001 | 85.04 | 3 | <0.001 | 351.62 | 3 | <0.001 |
| **Body mass** | 0.35 | 1 | 0.557 | 0.96 | 1 | 0.326 | 0.33 | 1 | 0.568 | 0.07 | 1 | 0.787 |
| **Developmental stage*organ** | 104.99 | 12 | <0.001 | 248.49 | 12 | <0.001 | 53.79 | 12 | <0.001 | 87.32 | 12 | <0.001 |
| **Citrate synthase activity** |  |  |  | 64.31 | 1 | <0.001 | 39.33 | 1 | <0.001 |  |  |  |

**Table S3.** Post-hoc tests testing differences in mitochondrial volume (citrate synthase activity) between developmental stages from metamorphosis to adulthood in the gut, liver, and hindlimb muscle of *Xenopus laevis*. Overall model for heart was not significant and thus post-hoc tests were not conducted. *P*-values are corrected with *false discovery rate* adjustment.

|  | **Gut** | | | | | **Liver** | | | | | **Hindlimb muscle** | | | | | |
| --- | --- | --- | --- | --- | --- | --- | --- | --- | --- | --- | --- | --- | --- | --- | --- | --- |
|  | **Est** | **SE** | **df** | **t-ratio** | ***P*-value** | **Est** | **SE** | **df** | **t-ratio** | ***P*-value** | | **Est** | **SE** | **df** | **t-ratio** | ***P*-value** |
| **60NF-66NF** | -0.63 | 0.33 | 12.00 | -1.84 | 0.026 | 0.35 | 0.13 | 13.45 | 2.75 | 0.032 | | -0.30 | 0.13 | 19.56 | -2.20 | 0.057 |
| **60NF-70d** | -0.83 | 0.33 | 12.20 | -2.48 | 0.072 | 0.18 | 0.13 | 13.37 | 1.42 | 0.223 | | -0.52 | 0.13 | 15.27 | -3.89 | 0.004 |
| **60NF-7m** | 0.25 | 0.33 | 13.40 | 0.77 | 0.644 | 0.06 | 0.12 | 14.18 | 0.49 | 0.631 | | -0.73 | 0.13 | 16.52 | -5.60 | <0.001 |
| **60NF-2y** | -0.77 | 0.35 | 13.40 | -2.23 | 0.087 | 0.71 | 0.13 | 15.21 | 5.43 | <0.001 | | -0.89 | 0.14 | 17.59 | -6.45 | <0.001 |
| **66NF-70d** | -0.21 | 0.32 | 22.58 | -0.66 | 0.644 | -0.17 | 0.12 | 23.81 | -1.41 | 0.223 | | -0.22 | 0.13 | 23.51 | -1.73 | 0.120 |
| **66NF-7m** | 0.87 | 0.32 | 21.32 | 2.70 | 0.045 | -0.29 | 0.12 | 21.54 | -2.44 | 0.039 | | -0.44 | 0.13 | 22.06 | -3.42 | 0.005 |
| **66NF-2y** | -0.16 | 0.34 | 22.42 | -0.46 | 0.720 | 0.36 | 0.12 | 26.72 | 2.90 | 0.019 | | -0.59 | 0.13 | 24.93 | -4.41 | <0.001 |
| **70d-7m** | 1.08 | 0.32 | 21.42 | 3.36 | 0.021 | -0.12 | 0.12 | 20.86 | -1.01 | 0.359 | | -0.21 | 0.13 | 20.28 | -1.68 | 0.120 |
| **70d-2y** | 0.06 | 0.34 | 20.59 | 0.17 | 0.867 | 0.52 | 0.12 | 22.26 | 4.21 | 0.001 | | -0.37 | 0.13 | 21.28 | -2.76 | 0.019 |
| **7m-2y** | -1.02 | 0.33 | 28.60 | -3.10 | 0.021 | 0.64 | 0.12 | 30.84 | 5.43 | <0.001 | | -0.16 | 0.13 | 33.29 | -1.23 | 0.227 |

**Table S4.** Post-hoc tests testing differences in oxidative capacity (succinate dehydrogenase activity) between developmental stages from metamorphosis to adulthood in the gut, liver, heart, and hindlimb muscle of *Xenopus laevis*. *P*-values are corrected with *false discovery rate* adjustment.

|  | **Gut** | | | | | **Liver** | | | | | **Heart** | | | | | | **Hindlimb muscle** | | | | | |
| --- | --- | --- | --- | --- | --- | --- | --- | --- | --- | --- | --- | --- | --- | --- | --- | --- | --- | --- | --- | --- | --- | --- |
|  | **Est** | **SE** | **df** | **t-ratio** | ***P*-value** | **Est** | **SE** | **df** | **t-ratio** | ***P*-value** | **Est** | **SE** | **df** | **t-ratio** | ***P*-value** | **Est** | | **SE** | **df** | **t-ratio** | ***P*-value** |  |
| **60NF-66NF** | 0.35 | 0.25 | 13.60 | 1.41 | 0.260 | 0.82 | 0.34 | 15.34 | 2.39 | 0.030 | 0.27 | 0.24 | 6.57 | 1.13 | 0.329 | -0.85 | | 0.24 | 19.24 | -3.58 | 0.020 |  |
| **60NF-70d** | -0.29 | 0.25 | 13.75 | -1.16 | 0.333 | 0.69 | 0.34 | 14.14 | 2.06 | 0.059 | -1.18 | 0.24 | 8.59 | -4.83 | 0.002 | -0.36 | | 0.26 | 24.91 | -1.42 | 0.242 |  |
| **60NF-7m** | -0.49 | 0.23 | 13.25 | -2.13 | 0.104 | 0.97 | 0.31 | 13.66 | 3.09 | 0.008 | -2.02 | 0.26 | 10.85 | -7.86 | <0.001 | -0.68 | | 0.28 | 24.84 | -2.44 | 0.075 |  |
| **60NF-2y** | -0.70 | 0.25 | 14.09 | -2.82 | 0.034 | 0.76 | 0.39 | 26.71 | 1.96 | 0.061 | -1.95 | 0.25 | 8.18 | -7.69 | <0.001 | -0.83 | | 0.30 | 30.54 | -2.80 | 0.044 |  |
| **66NF-70d** | -0.64 | 0.23 | 21.70 | -2.77 | 0.034 | -0.13 | 0.31 | 15.37 | -0.42 | 0.683 | -1.45 | 0.22 | 12.92 | -6.51 | <0.001 | 0.49 | | 0.22 | 18.85 | 2.20 | 0.084 |  |
| **66NF-7m** | -0.84 | 0.24 | 25.41 | -3.44 | 0.010 | 0.15 | 0.30 | 20.59 | 0.51 | 0.615 | -2.29 | 0.25 | 16.83 | -9.12 | <0.001 | 0.17 | | 0.24 | 17.17 | 0.72 | 0.532 |  |
| **66NF-2y** | -1.05 | 0.25 | 21.51 | -4.26 | 0.003 | -0.06 | 0.32 | 25.30 | -0.20 | 0.845 | -2.23 | 0.24 | 11.51 | -9.15 | <0.001 | 0.03 | | 0.26 | 21.70 | 0.10 | 0.924 |  |
| **70d-7m** | -0.20 | 0.24 | 25.12 | -0.81 | 0.424 | 0.28 | 0.30 | 17.71 | 0.94 | 0.361 | -0.84 | 0.24 | 20.42 | -3.53 | 0.003 | -0.32 | | 0.20 | 16.16 | -1.55 | 0.234 |  |
| **70d-2y** | -0.41 | 0.24 | 18.37 | -1.70 | 0.178 | 0.07 | 0.34 | 22.57 | 0.19 | 0.840 | -0.77 | 0.23 | 14.83 | -3.36 | 0.005 | -0.46 | | 0.21 | 16.47 | -2.20 | 0.084 |  |
| **7m-2y** | -0.21 | 0.25 | 31.52 | -0.83 | 0.424 | -0.22 | 0.34 | 42.84 | -0.65 | 0.521 | 0.07 | 0.23 | 17.10 | 0.30 | 0.766 | -0.15 | | 0.20 | 20.63 | -0.75 | 0.532 |  |

**Table S5.** Post-hoc tests testing differences in oxygen consumption capacity (cytochrome oxidase c) between developmental stages from metamorphosis to adulthood in the gut, liver, heart, and himdlimb muscle of *Xenopus laevis*. Only Post-hoc tests for liver and heart are represent since overall models were not significant for the gut and liver. *P*-values are corrected with *false discovery rate* adjustment.

|  |  |  | **Liver** |  |  |  |  | **Heart** |  |  |
| --- | --- | --- | --- | --- | --- | --- | --- | --- | --- | --- |
|  | **Est** | **SE** | **df** | **t-ratio** | ***P*-value** | **Est** | **SE** | **df** | **t-ratio** | ***P*-value** |
| **60NF-66NF** | 0.12 | 0.24 | 19.65 | 0.50 | 0.691 | 0.07 | 0.23 | 10.28 | 0.30 | 0.773 |
| **60NF-70d** | -0.28 | 0.22 | 13.76 | -1.26 | 0.311 | -0.98 | 0.24 | 12.19 | -4.11 | 0.004 |
| **60NF-7m** | -0.25 | 0.21 | 13.01 | -1.21 | 0.311 | -1.04 | 0.22 | 10.43 | -4.75 | 0.002 |
| **60NF-2y** | 0.52 | 0.26 | 25.68 | 1.97 | 0.161 | -0.66 | 0.23 | 11.04 | -2.84 | 0.027 |
| **66NF-70d** | -0.40 | 0.21 | 17.88 | -1.92 | 0.161 | -1.04 | 0.20 | 23.34 | -5.10 | <0.001 |
| **66NF-7m** | -0.37 | 0.21 | 22.62 | -1.73 | 0.161 | -1.11 | 0.20 | 16.22 | -5.65 | <0.001 |
| **66NF-2y** | 0.40 | 0.23 | 29.47 | 1.72 | 0.161 | -0.73 | 0.21 | 17.37 | -3.47 | 0.006 |
| **70d-7m** | 0.03 | 0.21 | 17.96 | 0.15 | 0.884 | -0.06 | 0.20 | 20.35 | -0.32 | 0.773 |
| **70d-2y** | 0.80 | 0.25 | 27.35 | 3.22 | 0.022 | 0.32 | 0.21 | 20.13 | 1.48 | 0.194 |
| **7m-2y** | 0.77 | 0.25 | 33.29 | 3.05 | 0.022 | 0.38 | 0.18 | 21.55 | 2.06 | 0.074 |

**Table S6.** Post-hoc tests testing differences in anaerobic capacity (lactate dehydrogenase) between developmental stages from metamorphosis to adulthood in the gut, liver, heart, and himdlimb muscle of *Xenopus laevis*. *P*-values are corrected with *false discovery rate* adjustment.

|  |  |  | **Gut** |  |  |  |  | **Liver** |  |  |  |  | **Heart** |  |  | **Hindlimb muscle** | | | | |
| --- | --- | --- | --- | --- | --- | --- | --- | --- | --- | --- | --- | --- | --- | --- | --- | --- | --- | --- | --- | --- |
|  | **Est** | **SE** | **df** | **t-ratio** | ***P*-value** | **Est** | **SE** | **df** | **t-ratio** | ***P*-value** | **Est** | **SE** | **df** | **t-ratio** | ***P*-value** | **Estimate** | **SE** | **df** | **t-ratio** | ***P*-value** |
| **60NF-66NF** | 0.72 | 0.27 | 60.98 | 2.61 | 0.028 | -0.38 | 0.16 | 60.84 | -2.33 | 0.040 | -0.28 | 0.19 | 54.99 | -1.51 | 0.153 | -0.81 | 0.19 | 59.83 | -4.16 | <0.001 |
| **60NF-70d** | -0.08 | 0.27 | 60.52 | -0.29 | 0.769 | -0.36 | 0.16 | 63.00 | -2.32 | 0.040 | -0.62 | 0.20 | 49.00 | -3.19 | 0.004 | -0.83 | 0.18 | 57.78 | -4.62 | <0.001 |
| **60NF-7m** | 0.14 | 0.25 | 57.42 | 0.58 | 0.629 | -0.68 | 0.15 | 61.65 | -4.57 | <0.001 | -1.46 | 0.19 | 54.24 | -7.69 | <0.001 | -1.77 | 0.17 | 54.06 | -10.54 | <0.001 |
| **60NF-2y** | -0.56 | 0.26 | 56.78 | -2.16 | 0.058 | -0.80 | 0.16 | 61.44 | -5.02 | <0.001 | -1.44 | 0.20 | 50.85 | -7.36 | <0.001 | -2.23 | 0.17 | 50.85 | -13.05 | <0.001 |
| **66NF-70d** | -0.80 | 0.25 | 58.05 | -3.14 | 0.013 | 0.02 | 0.15 | 61.42 | 0.12 | 0.903 | -0.34 | 0.18 | 53.66 | -1.95 | 0.071 | -0.02 | 0.17 | 54.92 | -0.11 | 0.911 |
| **66NF-7m** | -0.57 | 0.26 | 60.70 | -2.19 | 0.058 | -0.30 | 0.14 | 62.20 | -2.09 | 0.051 | -1.18 | 0.18 | 54.13 | -6.74 | <0.001 | -0.96 | 0.17 | 58.05 | -5.63 | <0.001 |
| **66NF-2y** | -1.28 | 0.28 | 60.85 | -4.54 | <0.001 | -0.42 | 0.16 | 62.23 | -2.60 | 0.029 | -1.16 | 0.18 | 53.42 | -6.43 | <0.001 | -1.42 | 0.19 | 59.20 | -7.66 | <0.001 |
| **70d-7m** | 0.22 | 0.25 | 58.44 | 0.90 | 0.463 | -0.32 | 0.15 | 61.83 | -2.20 | 0.045 | -0.84 | 0.18 | 50.04 | -4.61 | <0.001 | -0.95 | 0.16 | 53.32 | -6.04 | <0.001 |
| **70d-2y** | -0.48 | 0.27 | 60.82 | -1.76 | 0.118 | -0.44 | 0.16 | 62.96 | -2.74 | 0.027 | -0.81 | 0.18 | 53.41 | -4.48 | <0.001 | -1.40 | 0.17 | 56.14 | -8.23 | <0.001 |
| **7m-2y** | -0.71 | 0.26 | 59.39 | -2.71 | 0.028 | -0.12 | 0.15 | 61.54 | -0.79 | 0.479 | 0.03 | 0.18 | 54.91 | 0.14 | 0.885 | -0.46 | 0.16 | 51.89 | -2.86 | 0.007 |
